# Associations of rumination, behavioral activation, and perceived reward with mothers’ postpartum depression during the COVID-19 pandemic: a cross-sectional study

**DOI:** 10.3389/fpsyt.2024.1295988

**Published:** 2024-01-22

**Authors:** Miki Matsunaga, Junko Okajima, Kaichiro Furutani, Noriko Kusakabe, Nanako Nakamura-Taira

**Affiliations:** ^1^Department of Psychology, Rikkyo University, Niiza, Japan; ^2^Faculty of Informatics, Kansai University, Takatsuki, Japan; ^3^Department of Psychology, Faculty of Human Culture and Sciences, Fukuyama University, Fukuyama, Japan; ^4^Department of Psychology, Faculty of Letters, Chuo University, Hachioji, Japan

**Keywords:** postpartum depression, rumination, behavioral activation, reward perception, perfectionism, COVID-19

## Abstract

**Introduction:**

The COVID-19 pandemic has led to increased social isolation for mothers, and rumination exacerbates postpartum depression in mothers with poor social support. Although behavioral activation can help to decrease their depressive symptoms, the mechanism by which behavioral activation reduces postpartum depression remains unclear.

**Methods:**

We examined the effects of rumination and behavioral activation on depression in postpartum women by examining a model mediated by subjective reward perception. A questionnaire was administered to 475 postpartum women (Age: *Mean* = 30.74 years, *SD* = 5.02) within 1 year of childbirth using an Internet survey. The measurements included perinatal depression, rumination, and behavioral activation, and we assessed environmental reward. To control for confounding variables, we assessed psychiatric history, social support, parenting perfectionism, and COVID-19 avoidance.

**Results:**

Eighty-four (17.68%) mothers had possible postpartum depression. The covariance structure analysis showed that not only was there a direct positive path from rumination to postnatal depression but also a negative path via reward perception.

**Discussion:**

This finding indicated that the COVID-19 pandemic could have increased depression in many of the mothers. Rumination not only directly relates to postpartum depression, but it could also indirectly relate to postpartum depression by decreasing exposure to positive reinforcers. In addition, having a history of psychiatric illness increases the effect of rumination on postpartum depression. These findings suggest that psychological interventions are needed to reduce rumination and increase contact with positive reinforcements to reduce postpartum depression, especially for high-risk groups.

## 1 Introduction

Postpartum depression refers to depressive episodes that are prevalent following childbirth (1). Postpartum depression has major implications for the mother’s functioning, interpersonal relationships, parenting behaviors, and her offspring’s health and developmental outcomes (2, 3). A meta-analysis review showed a 12% incident rate of postpartum depression [95% confidence interval; CI: 0.04–0.20] among mothers (4), with a previous study in Japan showing a prevalence rate of 10%–15% (5). The spread of COVID-19 has led to increased social isolation during pregnancy and postpartum periods, with women avoiding interactions with others or not going out to prevent contracting the COVID-19 infection (6). Rates of perinatal depression and anxiety have thus increased during the COVID-19 pandemic (6–8).

However, the COVID-19 pandemic situation in Japan differed from that in other countries. Specifically, instead of implementing a lockdown of cities by law, the government requested self-restraint to reduce social interaction, along with hand hygiene and mask-wearing (9). Consequently, the traditional support system for perinatal women in Japan, “delivery at hometown,” was also restricted. This limitation left mothers vulnerable to the loss of social support and an increased burden of housework and childcare (10). Furthermore, as reported by Tsuno et al. (11), home visits by healthcare professionals and infant checkups or vaccinations were canceled. The deprivation of support during pregnancy or after delivery was also associated with postnatal depression.

According to the Diagnostic and Statistical Manual of Mental Disorders, Fifth Edition (12), perinatal depression involves a major depressive episode that occurs during pregnancy or within 4 weeks following delivery. Although the biological factors influencing mood during the early postpartum period may be less relevant subsequently, the first year after delivery is replete with many unique psychological stressors (13). In other clinical practice and research, postpartum depression is defined as occurring anytime within the first 12 months after childbirth (13, 14).

Ando and Muto (15) conducted a longitudinal study from the onset of pregnancy to 1 year postpartum. Factors influencing depression could differ depending on the time and physiological factors; for example, low estrogen levels could contribute to depression in the early postpartum period. Furthermore, from 6 months postpartum, the tendency to persist in self-directed emotions and attention, such as self-preoccupation, contributes to high depression levels. Maladaptive emotion regulation strategies (e.g., rumination, self-blame, and catastrophizing) have a significant negative effect on perinatal depression (16).

Regarding the relationship between rumination and postpartum depression, Raes et al. (17) reported that the level of rumination compared to negative affect did not predict depressive symptoms at 12 and 24 weeks postpartum when baseline symptoms and history of major depressive episodes were controlled. This result suggests that a history of major depression when pregnant or earlier is an important predictor of postpartum depression.

Additionally, O’Mahen et al. (18) found that women who were low in social functioning showed a higher correlation between rumination during pregnancy and postpartum depression. This suggests that social functioning moderates the impact of rumination on postpartum depression. In a later study, the researchers (19) also examined the impact of rumination on parental problem-solving effectiveness in postnatal mothers. They found that dysphoric ruminating mothers exhibited poorer problem-solving skills and less confidence regarding their problem-solving abilities compared to dysphoric distracting, non-dysphoric distracting, and non-dysphoric ruminating mothers.

As described above, rumination is associated with postpartum depression and it decreases postpartum mothers’ confidence in their ability to solve problems. Further, rumination exacerbates postpartum depression in mothers with a history of depression during or prior to pregnancy, and in those with poor social support.

Rumination is classified as an avoidance behavior, and it primarily involves cognitive avoidance coping. The behavioral theory posits that certain environmental changes and avoidant behaviors inhibit individuals from experiencing environmental rewards and reinforcement, subsequently leading to the development and maintenance of depressive symptoms.

Ferster (20) noted that the most obvious characteristic of a depressed person is the loss of interest in certain kinds of activities coupled with an increase in avoidance and escape activities such as complaints, crying, and remaining in bed all day. Such behaviors also reduce the frequency of behaviors that result from positive reinforcers. Additionally, owing to depressive symptoms, many of the depressed person’s behaviors do not receive sufficient reinforcement. As such, they have a high frequency of avoidance, escape from aversive stimuli, and have a reduced frequency of positively reinforced behavior. Consequently, they do not develop a repertoire of positive behaviors (problem-solving, goal achievement, etc.).

Carvalho and Hopko (21) examined the relationship between avoidance and depression using self-report and behavioral indices of environmental reward as proxy measures for positive reinforcement. They reported that both indices of environmental reward significantly mediated the relationship between avoidance and depression.

Thus, based on this behavioral model of depression, rumination is used as a means of coping with depressive mood and providing temporary mood relief, while maintaining and exacerbating the depressive mood in the long term because of reduced rewards.

Further, behavioral activation, such as increasing activity to increase exposure to rewards, is useful during depression. Increasing goal- and value-based activity levels reduces depression (22).

Regarding the postpartum (perinatal) period, women who received a 12-session guided Internet behavioral activation intervention showed a large effect size on depression at 6 months post-intervention compared with those in the treatment as usual group (23). Another study conducted behavioral activation for 8 weeks on postpartum mothers who were prone to isolation during the COVID-19 pandemic, using an online videoconferencing application [e.g., Zoom; (24)]. Participants reported that behavioral activation helped with support and social connection, creative problem-solving, and attending to pandemic-related symptoms.

Although evidence has shown that behavioral activation is effective for perinatal depression, the detailed mechanisms through which behavioral activation reduces postpartum depression via reward perception are unclear. Further, several risk factors for postpartum depression have been noted, in addition to behavioral factors such as rumination and behavioral activation. For example, the greatest predictor of postpartum depression was the assessment of psychiatric disorders both prior to and during pregnancy (25). Additionally, the strongest postpartum depression risk predictors among psychosocial factors were severe life events, some forms of chronic strain, relationship quality, and support from partners and mothers (26).

Perfectionism plays a critical role in anxiety disorders and depression (27). Definitions of perfectionism center on the pursuit of high standards and self-criticism over not meeting these standards. A meta-analysis showed that perfectionism was also associated with symptoms of maternal perinatal depression and anxiety (28). Parental perfectionism was positively correlated with parental burnout, and perfectionistic concerns were associated with increased tendencies to ruminate about the past (29).

**FIGURE 1:**
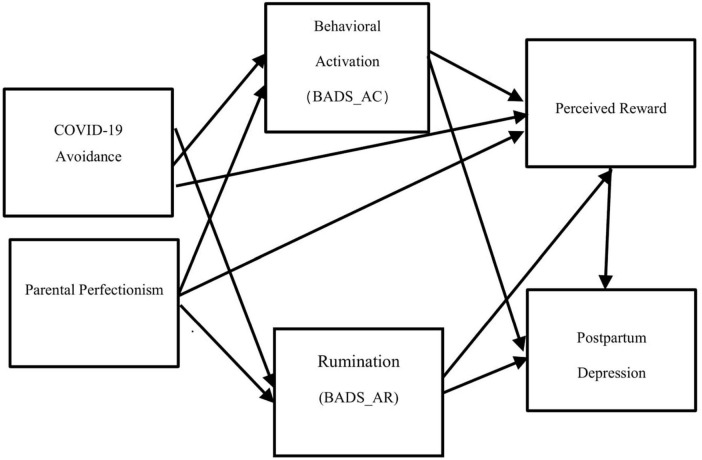
The hypothesized model. BADS, behavioral activation for depression scale; AC, activation factor; AR, avoidance/rumination factor; EROS, environmental reward observation scale; EPDS, Edinburgh postnatal depression scale.

We examined the effects of rumination and behavioral activation on depression in women within 1 year after childbirth by testing a model in which subjective reward perception acts as a mediator (Figure 1). We hypothesized that rumination would directly predict postpartum depression, which is predicted indirectly via reward perception. We expected that behavioral activation would directly reduce postpartum depression and indirectly reduce postpartum depression via reward perception. Additionally, we expected that avoiding closed and crowded situations to prevent COVID-19 infection and parenting perfectionism would affect postpartum depression via rumination, behavioral activation, and reward perception. We also tested whether the hypothesized model differs depending on risk factors for postpartum depression, such as a history of mental illness and support from partners or mothers, and clarify the exacerbating factors for postpartum depression in the high-risk group.

## 2 Materials and methods

### 2.1 Participant and procedures

We administered a survey registered with a web-based research firm, in January 2022, to 500 mothers. The inclusion criteria were mothers aged 18 years or older with a child under the age of 1. The exclusion criteria were women who were over 50 years old and could not read Japanese. Data from 475 mothers (valid response rate of 95%) were included in the analysis. However, data for mothers with more than one-third non-responses to all items (*n* = 13, 2.6%), with no children (*n* = 7, 1.4%), and with children older than 13 months (*n* = 5, 1.0%) were excluded. The mean age of the mothers was 30.74 years (*SD* = 5.02); most mothers were married (449, 94.53%) and on parental leave (279, 58.74%; Table 1).

**TABLE 1:** Participants’ characteristics.

Characteristic	Number of Women (*N* = 475)
Age (years)	30.74 (SD = 5.02)
**Marital status**	
Single	26 (5.47%)
Married	449 (94.53%)
**Number of children**	
1 (first pregnancy)	268 (56.42%)
2	144 (30.32%)
3	50 (10.53%)
4 or more	13 (2.74%)
Age of child (months)	6.04 (SD = 3.46)
**Sex of child**	
Boy	251 (52.84%)
Girl	220 (46.32%)
No answer	4 (0.84%)
**Work status**	
Not working	144 (30.32%)
Working	50 (10.53%)
Maternity leave	279 (58.74%)
**History of mental illness**	
Yes	65 (13.68%)
No	410 (86.32%)
**Support from partner**	
Yes	374 (78.74%)
No	92 (19.37%)
No partner	9 (1.89%)
**Support from own mother**	
Yes	332 (69.89%)
No	118 (24.84%)
No own mother	25 (5.26%)

Means are presented for continuous variables (with standard deviations in parentheses). Frequencies are presented for categorical variables.

Most children were from first-time mothers (268, 56.42%), and children’s mean age was 6.04 months (*SD* = 3.46). Children’s sex included 251 (52.84%) boys and 220 (46.32%) girls, while 4 (0.84%) mothers did not provide an answer.

The Research Ethics Committee of the Faculty of Contemporary Psychology at XXX University approved this study (no. ZZZ) [blinded for review]. Informed consent for use of data collected via questionnaires was obtained from participants following the protocol approved by the university’s research ethics committee.

### 2.2 Measures

The Edinburgh Postnatal Depression Scale (EPDS; (30)) was developed to screen for postpartum depression. Okano (31) developed the Japanese version of the EPDS that was used in this study. Participants were requested to respond to 10 questions about their feelings over the past 7 days based on a four-point scale (0–3). The total scores range from 0 to 30, with higher scores indicating a higher likelihood of postpartum depression. For the Japanese version, a score of nine or higher is regarded as indicative of probable depression. The sensitivity was 0.75, and the specificity was 0.93 in the study by Okano (31).

The cutoff point of nine for the Japanese version of the EPDS has been validated in other previous studies (32–34). The sensitivity and specificity of the Japanese version of the EPDS for identifying major depressive episodes both exceed 80%. The factor analysis of the Japanese version of EPDS has not been fully elucidated. First, a confirmatory factor analysis was conducted assuming a one-factor structure. However, the variables of goodness of fit were not sufficient (adjusted goodness of fit index; AGFI = 0.766, comparative fit index; CFI = 0.782, root mean square error of approximation; RMSEA = 0.165). Kubota et al. (35) reported that the result of exploratory factor analysis indicated a three-factor model consisting of anxiety, depression, and anhedonia. Therefore, a confirmatory factor analysis of three factors was conducted, and the goodness of fit was better than that of a one-factor structure; the statistical values were sufficient (AGFI = 0.952, CFI = 0.932, RMSEA = 0.080). These results supported the validity of the EPDS in this study. In addition, the EPDS was analyzed using the total score in accordance with previous studies (17–19). In this study, Cronbach’s alpha was 0.85.

The Japanese version of the Behavioral Activation for Depression Scale [BADS; (36)] measures the activation or avoidance of behavior and resulting impairment of social functioning. This scale comprises 25 items and four factors: Activation (BADS-AC), Avoidance/Rumination (BADS-AR), Work/School Impairment, and Social Impairment. The original version of the BADS was developed by Kanter et al. (37) who reported a good factor structure, Cronbach’s α = 0.87, and test–retest reliability coefficient of *r* = 0.74. The Japanese version has also demonstrated a good factor structure (RMSEA = 0.77), good internal consistency (α = 0.78), and good construct reliability. In the current study, the confirmatory factor analysis for the four-factor structure revealed that the goodness of fit was sufficient (AGFI = 0.779; CFI = 0.822, RMSEA = 0.090), and Cronbach’s alpha in this sample was 0.88, In this study, only the BADS-AC and AR subscales were used in the analysis to examine the relationship between behavioral activation and rumination, and postpartum depression. BADS-AC consists of seven items that assess not only one’s engagement in more pleasurable activities but rather in those that help them achieve their specific goals (37). BADS-AR consists of eight items that measure one’s avoidance of aversive thoughts and feelings. Kanter et al. (37) reported that the correlation between BADS-AR and the rumination subscale of the Response Styles Questionnaire was high (*r* = 0.64, *p* < 0.01). There was also a high correlation between BADS-AR and BDI (*r* = 0.63, *p* < 0.01). Previous studies have suggested that rumination predicts the onset, length, and severity of depressive episodes (38–40). In addition, Kanter et al. (37) proposed that rumination represents a dysfunctional attempt to avoid a depressive affect. In this study, participants rated each item on a seven-point scale ranging from 0 (*not at all*) to 6 (*completely applicable*), and scores were calculated for each factor. Higher scores indicate stronger characteristics for each factor.

The Japanese version of the Environmental Reward Observation Scale [EROS; (41)] comprises 10 items regarding the subjective evaluation of the level to which positive reinforcers (rewards) accompany one’s behavior. Four responses were requested (from 1 = *strongly disagree* to 4 = *strongly agree*). Five items were reverse scored. The higher the score, the higher the perceived reward associated with the behavior. Kunisato et al. (41) reported a good factor structure (AGFI = 0.90, CFI = 0.89, RMSEA = 0.08), good internal consistency (α = 0.78), and good test–retest reliability (*r* = 0.75) of the scale. In this study, a confirmatory factor analysis also showed that the goodness of fit was acceptable (AGFI = 0.772, CFI = 0.788, RMSEA = 0.133). In addition, Cronbach’s alpha was 0.79.

Avoidance of Cs from the classification of coping behaviors for COVID-19 was used (42). The World Health Organization proposed the three Cs based on English acronyms meaning “crowded places,” “close-contact settings,” and “confined and enclosed space” to prevent the spread of COVID-19. The re-spread of COVID-19 during this survey period (January 2022) was assumed to affect the avoidance of the Cs. Therefore, the three items of “avoidance of the three Cs” from Koiwa et al. (42) were used for nurses’ coping behaviors related to the COVID-19 infection. This scale assesses the degree of avoidance of the three Cs using a six-point scale (from 1 = *not at all* to 6 = *very applicable*). The coefficient was α = 0.85, indicating high consistency (42). Cronbach’s alpha in our sample was 0.86.

Parental perfectionism was evaluated (29). Participants were requested to respond to four questions about their tendency toward perfectionism in parenting based on a six-point scale ranging from 1 (*not at all*) to 6 (*very much*). Higher scores indicate a higher tendency toward perfectionism in parenting. A high reliability of this scale (α = 0.92) was reported (29). Cronbach’s alpha in our sample was 0.84.

The Childcare Support Checklist was used (43). Of the nine items, seven were used to assess childcare environmental factors, such as a history of mental illness, life events, housing and financial situation, support from one’s husband and own mother, and intimate interpersonal relationships. Two items concerned situations in which the mother was stuck in actual childcare situations and the feelings she had. Participants were requested to respond “yes” or “no” to each item.

### 2.3 Statistical analysis

The age and marital status of each participant were provided by an Internet research firm. The number of children, children’s age and gender, and employment status were asked in a sociodemographic questionnaire. Mental illness history, partner support, and support from the biological mother were asked through a parenting support checklist (43). Statistical analyses were conducted using SPSS 25.0 and AMOS 25.0 (IBM, Armonk, NY, USA). Descriptive statistics were computed for the demographic characteristics. The reliability of the measures was calculated using Cronbach’s alpha. In addition, we conducted a confirmatory factor analysis of the EPDS, BADS, and EROS using all items of the scales to examine their validity for the study population.

To test the basic relationships between variables, including sociodemographic variables, we computed Pearson’s correlations. Subsequently, a structural analysis of covariance was conducted to test the hypothesis that daily exposure to rewards (EROS) mediates the effect of behavioral activation (BADS_AC) and rumination (BADS_AR) on postpartum depression (as measured by the EPDS). To control for confounding variables, we assessed psychiatric history, social support, parenting perfectionism, and COVID-19 avoidance. This model also included the variables of avoidance of crowding and closeness because of the COVID-19 pandemic (avoidance of three Cs) and parental perfectionism. Further, we conducted a simultaneous multi-population analysis of the same model, dividing the groups according to whether they had a history of mental illness and the degree of perceived support from their partners and mothers, as measured using the Childcare Support Checklist (43).

## 3 Results

We calculated the means and standard deviations of the total scores for each variable and alpha coefficients. The alpha coefficients ranged from 0.76 to 0.85, confirming a high degree of internal consistency (Supplementary Appendix 1).

For the EPDS, 169 (35.58%) participants scored above the cutoff of nine points on the Japanese version.

Regarding Pearson’s correlation coefficients among sociodemographic assessment items and scales, there was no significant correlation between the EPDS scores and mother’s and children’s age. In addition, child’s sex, number of children, and work status had no significant correlation; however, there was a negative correlation between marital status and the EPDS (*r* = −0.081, *p* < 0.10), BADS-AR (*r* = −0.109, *p* < 0.05), perfectionism (*r* = −0.131, *p* < 0.01). These scores among unmarried mothers was higher (Supplementary Appendix 2).

The Pearson’s correlation coefficients among the measures indicated that all variables except for the three Cs related to COVID-19 avoidance significantly correlated with the EPDS. Higher levels of postpartum depression moderately correlated with higher levels of avoidance/rumination (*r* = 0.535), parental perfectionism (*r* = 0.378), and lower levels of perceived rewards (*r* = −0.560). Behavioral activation weakly correlated with postpartum depression (*r* = −0.243). Further, the correlation between perceived reward and behavioral activation was moderately positive (*r* = 0.478), and the correlation between perceived award and avoidance/rumination was weakly negative (*r* = −0.342). The association between parental perfectionism and avoidance/rumination was moderately positive (*r* = 0.460), and the association between parental perfectionism and perceived rewards was negatively associated (*r* = −0.391; all *p*s < 0.01).

### 3.1 Testing the hypothesized model

We conducted a covariance structure analysis to test whether behavioral activation (BAD_AC) and avoidance/rumination (BADS_AR) affected postpartum depression directly, or whether this relationship was mediated by reward perception (EROS). We also hypothesized that the paths of BADS_AC, BADS_AR, and EROS are related between parenting perfectionism and coping behaviors of COVID-19 infection. However, the model did not fit the data [χ^2^(3) = 29.547, *p* < 0.001, GFI = 0.980, AGFI = 0.861, CFI = 0.964, and RMSEA = 0.137].

Next, based on the correlation analysis results, we tested the modified version of the hypothesized model by adding a path of covariance between BADS_AC and BADS_AR and removing non-significant paths from the three Cs for COVID-19 avoidance and parenting perfectionism. The modified model had an excellent fit to the data [χ^2^(5) = 2.842, ns., GFI = 0.998. AGFI = 0.992, CFI = 1.000, and RMSEA = 0.000], and we referred to it as the final model.

**FIGURE 2:**
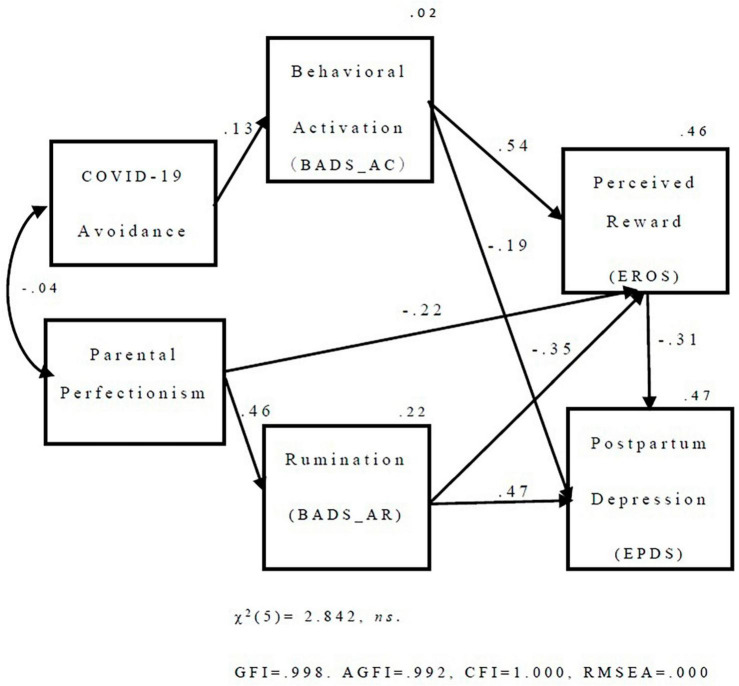
The results of covariance structure analysis. BADS, behavioral activation for depression scale; AC, activation factor; AR, avoidance/rumination factor; EROS, environmental reward observation scale; EPDS, Edinburgh postnatal depression scale.

The following paths were observed in the final model (Figure 2): a negative path (β = −0.19) from behavioral activation (BADS_AC) to postpartum depression (EPDS) and a positive path directly to reward perception (EROS; β = 0.54). In addition, a positive path (β = 0.47) directly from avoidance/rumination (BADS_AR) to postpartum depression (EPDS) and a negative path to reward perception (EROS; β = −0.35) were observed. The negative path from reward perceptions to postpartum depression (β = −31) and behavior activation and avoidance/rumination could affect postpartum depression mediated by reward perception. A positive path from parenting perfectionism to avoidance/rumination (β = 0.46) and a negative path to reward perception (β = −0.22) were observed. A positive path from the coping behavior of COVID-19 to behavior activation was also observed (β = 0.13).

### 3.2 Simultaneous multi-population analysis

We conducted a simultaneous multi-population analysis of risk factors for postpartum depression, such as mental illness history and support from partners and biological mothers.

The simultaneous multi-population analysis results showed that the GFI without equality restrictions was more appropriate regardless of the presence or absence of a history of mental illness (Table 2). Comparing the path coefficients for the two groups, the path from avoidance/rumination to reward perception was not significant for mothers with a history of mental illness and only the path directly affecting depression was significant. Conversely, the direct path from behavioral activation to depression was not significant; only the path affecting depression via reward perception was significant. Among mothers with no history of mental illness, the positive path from the three Cs for COVID-19 avoidance to behavior activation was significant, whereas this path was not significant for those mothers with a history of mental illness.

**TABLE 2:** Goodness-of-fit and internal assessment of the simultaneous multi-population analysis model in mothers with or without a history of mental illness.

Goodness-of fit indexes GFI = 0.993, AGFI = 0.977, CFI = 1.000, RMSEA = 0.000, AIC = 69.706				
Variable	Mothers without a history of mental illness (*n* = 410)		Mothers with a history of mental illness (*n* = 65)	
	Standardized estimates	*p*	Standardized estimates	*p*
COVID-19 Avoidance→BADS_AC	0.163	<0.001	-0.102	0.396
Parental Perfectionism→BADS_AR	0.418	<0.001	0.542	<0.001
Parental Perfectionism→EROS	0.193	<0.001	-0.378	0.002
BADS_AC→EROS	0.576	<0.001	0.405	<0.001
BADS_AR→EROS	-0.345	<0.001	-0.152	0.211
EROS→EPDS	-0.287	<0.001	-0.243	0.018
BADS_AC→EPDS	-0.213	<0.001	-0.165	0.101
BADS_AR→EPDS	0.425	<0.001	0.590	<0.001

BADS, behavioral activation for depression scale; AC, activation factor; AR, avoidance/rumination factor; EROS, environmental reward observation scale; EPDS, Edinburgh postnatal depression scale.

We also conducted simultaneous multi-population analyses for support from partners (yes/no) and biological mothers (yes/no). In both analyses, the GFI without equality restrictions was more appropriate than that with equality restrictions. Regarding the presence of partners’ support, there was a significant group difference in the value of the path from the three Cs for COVID-19 avoidance to behavior activation. In the group that did not expect support from their partners, there was no significant association between avoidance of crowding and closeness because of the COVID-19 pandemic and behavior activation (Table 3).

**TABLE 3:** Goodness-of-fit and internal assessment of the simultaneous multi-population analysis model in mothers with or without their partner’s support.

Goodness-of fit indexes GFI = 0.987, AGFI = 0.955, CFI = 0.988, RMSEA = 038, AIC = 80.296				
Variable	Mothers with their partner’s support (*n* = 374)		Mothers without their partner’s support (*n* = 101)	
	Standardized estimates	*p*	Standardized estimates	*p*
COVID-19 Avoidance→BADS_AC	0.171	<0.001	-0.026	0.788
Parental Perfectionism→BADS_AR	0.406	<0.001	0.538	<0.001
Parental Perfectionism→EROS	-0.212	<0.001	-0.251	0.001
BADS_AC→EROS	0.518	<0.001	0.638	<0.001
BADS_AR→EROS	-0.339	<0.001	-0.313	<0.001
EROS→EPDS	-0.286	<0.001	-0.288	0.006
BADS_AC→EPDS	-0.196	<0.001	-0.190	0.059
BADS_AR→EPDS	0.456	<0.001	0.500	<0.001

BADS, behavioral activation for depression scale; AC, activation factor; AR, avoidance/rumination factor; EROS, environmental reward observation scale; EPDS, Edinburgh postnatal depression scale.

Regarding biological mothers’ support, there were significant group differences in the values of the paths from parenting perfectionism to avoidance/rumination (*p* < 0.01), behavior activation to reward perception (*p* < 0.01), and avoidance of crowding and closeness for the prevention of COVID-19 to behavior activation (*p* < 0.05). In the group that did not expect support from their biological mothers, there was no significant association between avoidance of crowding and closeness because of the COVID-19 pandemic and behavior activation (Table 4).

**TABLE 4:** Goodness-of-fit and internal assessment of the simultaneous multi-population analysis model in mothers with or without their own mother’s support.

Goodness-of fit indexes GFI = 0.991, AGFI = 0.968, CFI = 0.998, RMSEA = 0.014, AIC = 73.080				
Variable	Mothers with their own mother’s support (*n* = 332)		Mothers without their own mother’s support (*n* = 143)	
	Standardized estimates	*p*	Standardized estimates	*p*
COVID-19 Avoidance→BADS_AC	0.200	<0.001	-0.017	0.839
Parental Perfectionism→BADS_AR	0.381	<0.001	0.569	<0.001
Parental Perfectionism→EROS	-0.221	<0.001	-0.227	<0.001
BADS_AC→EROS	0.500	<0.001	0.622	<0.001
BADS_AR→EROS	-0.326	<0.001	-0.379	<0.001
EROS→EPDS	-0.302	<0.001	-0.318	<0.001
BADS_AC→EPDS	-0.168	<0.001	-0.231	0.004
BADS_AR→EPDS	0.469	<0.001	0.475	<0.001

BADS, behavioral activation for depression scale; AC, activation factor; AR, avoidance/rumination factor; EROS, environmental reward observation scale; EPDS, Edinburgh postnatal depression scale.

## 4 Discussion

This study examined the associations between avoidance/rumination and postpartum depression, and between behavioral activation and postpartum depression, among women within 1 year of childbirth during the COVID-19 pandemic. Further, we examined whether these behavioral patterns affect postpartum depression via reward perception and whether risk factors for postpartum depression, such as a history of mental illness and partner and biological mother’s support, differed in the hypothesized model. Additionally, we clarified the factors that were associated with postpartum depression in the high-risk group.

This study’s results revealed the following:

A tendency toward postnatal depression was observed in approximately 35% of the participants. This finding indicates that the COVID-19 pandemic could have increased loneliness and depression in many mothers.

Rumination was directly related to postpartum depression and could have increased it by decreasing exposure to positive reinforcers.

Parenting perfectionism could be related to rumination and associated postpartum depression by decreasing the perception of and contact with positive reinforcers.

For mothers with a history of mental illness, emotion regulation had a greater impact on postpartum depression compared to those mothers without a history of mental illness. Additionally, the approach to prevent COVID-19 infection by avoiding the three Cs led to behavioral inactivity in mothers with a history of mental illness.

Although previous researchers have reported that rumination is significantly associated with postpartum depression, this is the first study to indicate that rumination is related to postpartum depression via a decrease in positive reinforcers.

Recent laboratory studies on rumination and reward perception showed that rumination disrupted reinforcement learning (adjusting behavior after error to behavior), thereby impairing learning adaptive behavior and promoting stress-generating behavior; however, participants were not in their perinatal period (44).

Rumination might impair concentration and contingency attention associated with adaptive behavior, suggesting that rumination reduces sensitivity to contextual details (45). Further, a study on experimental rumination manipulations with postpartum mothers reported that maternal sensitivity toward infants significantly decreased after induction of the rumination task (46).

In summary, excessive rumination by postpartum mothers about their mood and consequences of their behaviors could further impair adaptive behaviors by making it difficult for them to focus on adaptive behaviors and reducing maternal sensitivity to their infants. For example, excessive ruminative thoughts could reduce their focus on successful parenting or lead to a decrease in opportunities to be in-touch with positive reinforcers because of the inability to respond to their infants’ cries and actions.

Conversely, behavioral activation might indirectly reduce postpartum depression through the perception of positive reinforcers. This result indicates that both being active and increasing exposure to positive reinforcers are necessary to prevent postpartum depression. Particularly among mothers with a history of mental illness, the direct path from behavioral activation to postpartum depression was not significant, whereas the indirect path via reward perception was significant. This suggests the importance of increasing activities that match their values in life and increase opportunities to perceive positive reinforcement through parenting behaviors rather than simply extending their activities. Further, the difference in the values of the paths from behavioral activation to perceived reward was significantly higher for mothers who did not expect support from their own mothers compared to those who had their mothers’ support. This finding suggests that an increased exposure to positive reinforcement through their behavior could reduce postpartum depression in mothers who do not expect support from their own mothers.

This survey was conducted during the COVID-19 pandemic, and approximately 36% of the total population had an EPDS score of 9 or higher, as the cutoff score for the Japanese version of the EPDS. The rate is higher than the positive screening rate of 14% (47) or 29% for postpartum depression (11) in Japan during the pandemic. However, other studies did not report this difference, and whether there was any difference in the prevalence of postnatal depressive symptoms between the pre- and current COVID-19 periods could not be confirmed. The reason this cannot be confirmed is because of the variation in each country’s state of emergency. The unclear classification of pre-COVID-19 and COVID-19 cohorts could have led to ambiguous findings and could not provide an accurate point of comparison, compared to a pooled prevalence reported in previously published reviews. Although a state of emergency was declared in the Tokyo metropolitan area and other urban areas during this study period, determining whether the spread of COVID-19 increased the rate of postpartum depression more than usual was difficult to determine. This is because this study included mothers from areas in which emergency declarations had not been issued and more than 1 year had passed since the COVID-19 pandemic began.

The tendency to avoid the three Cs because of the COVID-19 pandemic had a positive effect on the activation of usual behavior. This result indicates that the behavior of coping with the fear of COVID-19 infection could not necessarily be predictive of postpartum depression, as mothers are usually goal-oriented in their behavior. However, for mothers with a history of mental illness and those who could not expect support from their partners or their own mothers, the path from avoidance behavior of COVID-19 infection to usual behavioral activation was negative but not significant. Nevertheless, the possibility that these behavioral restrictions caused by the COVID-19 pandemic could increase postpartum depression cannot be completely ruled out.

Regarding the relationship between perfectionism and rumination, the results indicated that mothers who had a strong tendency toward perfectionism in parenting were more likely to ruminate and less likely to perceive rewards in their daily lives. A longitudinal study of the relationship between perfectionism and repetitive negative thinking (RNT) in antenatal and postnatal mothers found that antenatal perfectionism predicted antenatal RNT and RNT was associated with antenatal depression (48) Additionally, this longitudinal study revealed that antenatal perfectionism affects postpartum depression via RNT and antenatal depression.

Although antenatal conditions were not examined, the results are consistent with the findings of Egan et al. (48)—that parenting perfectionism affects postpartum depression via rumination. A meta-analysis on perfectionism and postpartum depression also indicates that the strength of the relationship between perfectionism and postpartum depression could increase with the length of the postpartum period (49). Shafran et al. (50) suggested that individuals with perfectionism are self-critical and evaluate themselves negatively after failing to meet their standards. Although their standards were achievable, they might have considered them as insufficiently demanding.

Based on the above information, we assume that mothers with strong parenting perfectionism might compulsively be perfect in caring for their infants and ruminate when they fail to meet their demanding standards. Even when they meet their demanding standards, they tend to discount the consequences of their behavior and could experience fewer reinforcers or have a reduced sense of positive reinforcers accompanying their behaviors. Supporting mothers with strong parenting perfectionism in adjusting their parenting goals appropriately could prove useful. This would change their cognition and behaviors: avoid blaming themselves for their mistakes or failures, realize that no mother can do everything from the beginning, and accept that becoming a mother entails learning through trial and error.

### 4.1 Limitations

The current results indicate that rumination was not only directly associated with postpartum depression but also indirectly linked with it via perceived reward. However, several issues remain to be addressed in future studies. First, the relationship between rumination, behavior activation, and postpartum depression before the COVID-19 pandemic should be reexamined. Regarding behavior, COVID-19 infection reduced opportunities for interpersonal interactions and going outdoors. This study considered this by including the use of measurements that assess the avoidance of crowded places, confined and enclosed spaces, and close-contact settings as variables in the analysis; however, other coping behaviors toward COVID-19 were not assessed. Further, the activity level itself could have decreased during this period, which might have also influenced the current results. In addition, we did not measure the presence or absence of previous infection with COVID-19, hospitalization, or vaccination status during pregnancy in this study. Owing to the reduction in the activity level, the rate of COVID-19 infection among pregnant women in Japan was reportedly significantly lower at 0.6% (51) than in other countries. For instance, in the US, the rate of positive test results for SARS-CoV-2 among pregnant women was 6.6% (52). Based on this observation, we hypothesized that coping behaviors aimed at preventing infection (COVID-19 avoidance), such as limiting social interactions, were more influential in causing postpartum depression than the presence of COVID-19 infection.

Second, factors related to the children were not measured. Leigh and Milgrom (53) identified that risk factors, including low self-esteem, low social support, negative cognitive attributional style, concomitant high anxiety in pregnancy, and major life events such as low income and a history of childhood sexual abuse mostly predict three outcomes: antenatal depression, postnatal depression, and parenting stress—as well as the relationship between them. Fredriksen et al. (54) also reported that parenting stress mediates the relationship between parental depressive symptoms and child developmental outcomes. Therefore, future studies should consider factors related to children and parenting stress to clarify the relationship between rumination, reward perception, and postpartum depression. Finally, the design of this study was cross-sectional; thus, it was not possible to draw conclusions about the causal relationship and true mediation of each variable. Furthermore, all variables were measured by self-report from the same informant. It was easier to find associations between variables reported by the same participant, inflating the effect size. For EPDS, the proportion of mothers with possible depression may have been overestimated because self-report measures were used to assess depressive symptoms and a structured diagnostic interview with a clinician was not performed (55) In addition, depressed individuals were negatively biased in their reports of internal status and past events.

## 5 Conclusion

Rumination and behavior activation not only affected postpartum depression directly but also indirectly through perceived rewards among postpartum women who gave birth during the COVID-19 pandemic. The hypothesized model also differed according to risk factors for postpartum depression, such as the presence or absence of a history of mental illness and support from partners and mothers. From a clinical perspective, these findings suggest that as with general depression, it could be useful to use behavioral activation to reduce preoccupation with rumination and increase contact with and perception of positive reinforcers to reduce postpartum depression. Especially for groups at high risk of postpartum depression, it is necessary to provide support and praise for what has been done so that they can perceive positive reinforcement.

In addition, for parenting perfectionism, it is possible to change high demanding standards to appropriate ones through cognitive behavioral strategies, such as cognitive restructuring and behavioral experiments, and decrease self-blame for mistakes and failures to reduce postpartum depression.

## Data availability statement

The raw data supporting the conclusions of this article will be made available by the authors, without undue reservation.

## Ethics statement

The studies involving humans were approved by the Research Ethics Committee of the Faculty of Contemporary Psychology at Rikkyo University. The studies were conducted in accordance with the local legislation and institutional requirements. Written informed consent for participation was not required from the participants or the participants’ legal guardians/next of kin because it was an unnamed survey using a web-based survey company.

## Author contributions

MM: Conceptualization, Data curation, Formal analysis, Funding acquisition, Investigation, Methodology, Project administration, Visualization, Writing – original draft, Writing – review and editing. JO: Conceptualization, Data curation, Investigation, Methodology, Visualization, Writing – original draft, Writing – review and editing. KF: Data curation, Formal analysis, Investigation, Methodology, Writing – original draft, Writing – review and editing. NK: Conceptualization, Data curation, Methodology, Writing – original draft, Writing – review and editing. NN-T: Conceptualization, Data curation, Methodology, Writing – original draft, Writing – review and editing.
