# Supplementary material for: Associations of rumination, behavioral activation, and perceived reward with mothers’ postpartum depression during the COVID-19 pandemic: a cross-sectional study
Source: Front Psychiatry. 2024 Jan 22;15:1295988. doi: 10.3389/fpsyt.2024.1295988 (PMC10838984; doi:10.3389/fpsyt.2024.1295988)
Supplement: Supplementary file 1 [file Table_1.DOCX]

Supplementary Material

Associations of Rumination, Behavioral Activation, and Perceived Reward with Mothers’ Postpartum Depression During the COVID-19 Pandemic: A Cross-Sectional Study

**Miki Matsunaga, Junko Okajima, Kaichiro Furutani, Noriko Kusakabe, Nanako Nakamura-Taira**

Miki Matsunaga
mikim@rikkyo.ac.jp

Appendix 1 Descriptive statistics for variables in the model

|  | M | SD | α |
| --- | --- | --- | --- |
| EPDS | 7.11 | 5.47 | 0.85 |
| BADS_AC | 23.75 | 8.10 | 0.84 |
| BADS_AR | 21.09 | 8.37 | 0.84 |
| EROS | 27.22 | 4.96 | 0.79 |
| Coping Behavior on Covid-19 | 13.33 | 3.65 | 0.89 |
| Parental Perfectionism | 8.61 | 4.53 | 0.84 |

Appendix 2 Correlation coefficient matrix

|  | 1 |  | 2 |  | 3 |  | 4 |  | 5 |  | 6 |  | 7 |  | 8 |  | 9 |  | 10 |  | 11 |
| --- | --- | --- | --- | --- | --- | --- | --- | --- | --- | --- | --- | --- | --- | --- | --- | --- | --- | --- | --- | --- | --- |
| 1. Mother’s age | 1.000 |  |  |  |  |  |  |  |  |  |  |  |  |  |  |  |  |  |  |  |  |
| 2. Children’s age | .156 | ** | 1.000 |  |  |  |  |  |  |  |  |  |  |  |  |  |  |  |  |  |  |
| 3. Number of children | .227 | ** | -.033 |  | 1.000 |  |  |  |  |  |  |  |  |  |  |  |  |  |  |  |  |
| 4. Child’s sex | .022 |  | -.068 |  | .060 |  | 1.000 |  |  |  |  |  |  |  |  |  |  |  |  |  |  |
| 5. Work status | .097 | * | -.062 |  | -.042 |  | -.031 |  | 1.000 |  |  |  |  |  |  |  |  |  |  |  |  |
| 6. Marital status | .045 |  | .062 |  | .087 | + | -.081 | + | .025 |  | 1.000 |  |  |  |  |  |  |  |  |  |  |
| 7. EPDS | -.075 |  | .007 |  | .009 |  | -.034 |  | -.028 |  | -.081 | + | 1.000 |  |  |  |  |  |  |  |  |
| 8. BADS_AC | -.005 |  | -.069 |  | -.063 |  | .101 | * | .018 |  | -.045 |  | -.243 | ** | 1.000 |  |  |  |  |  |  |
| 9. BADS_AR | -.073 |  | -.033 |  | .032 |  | .029 |  | .101 | * | -.109 | * | .535 | ** | .198 | ** | 1.000 |  |  |  |  |
| 10. EROS | -.001 |  | -.078 | + | -.112 | * | .042 |  | -.005 |  | .054 |  | -.560 | ** | .478 | ** | -.342 | ** | 1.000 |  |  |
| 11. COVID-19 Avoidance | .007 |  | -.030 |  | .051 |  | -.083 | + | .049 |  | .073 |  | -.070 |  | .130 | ** | -.012 |  | .059 |  | 1.000 |
| 12. Parental Perfectionism | -.112 | * | .019 |  | .008 |  | .035 |  | -.002 |  | -.131 | ** | .378 | ** | -.025 |  | .460 | ** | -.391 | ** | -.041 |

Note: + *p* < .10, * *p* < .05, ** *p* < .01

BADS, Behavioral Activation for Depression Scale; AC, Activation factor; AR, Avoidance/Rumination factor; EROS, Environmental Reward Observation Scale; EPDS, Edinburgh Postnatal Depression Scale.
